# Social determinants of health-based strategies to address vaccination disparities through a university-public health partnership

**DOI:** 10.1017/cts.2024.502

**Published:** 2024-04-01

**Authors:** Susie Crowe, Carlyn Kimiecik, Omolola A. Adeoye-Olatunde, Megan Conklin, Jordan Smith, Sonak D. Pastakia, Alicia Dinkeldein, Mary Dubinin, Peter Zubler, Jasmine D. Gonzalvo

**Affiliations:** 1 Center for Health Equity and Innovation, Purdue University, College of Pharmacy, Indianapolis, IN, USA; 2 Walgreens Co, Indianapolis, IN, USA; 3 Wheeler Mission Lighthouse Center, Indianapolis, IN, USA; 4 Gleaners Food Bank of Indiana, Indianapolis, IN, USA; 5 St. Vincent de Paul. Indianapolis, IN, USA

**Keywords:** Evaluation, partnerships, social determinants of health, vaccines, vaccine disparities

## Abstract

A decline in routine vaccinations, attributed to vaccine hesitancy, undermines preventative healthcare, impacting health and exacerbating vaccine disparities. University-public health partnerships can improve vaccination services. This study describes and evaluates a university-public health use case employing social determinants of health (SDoH)-based strategies to address vaccination disparities. Guided by the Translational Science Benefits Logic Model, the partnership offered no-cost preventative vaccines at community-based organization (CBO) sites, collected CBO clientele’s vaccination interest, hesitancy, and demographic data, and conducted descriptive analyses. One hundred seven vaccination events were held, administering 3,021 vaccines. This partnership enhanced health outcomes by addressing disparities through co-located vaccination and SDoH services.

Vaccine disparities and preventative healthcare are critical in public health efforts and support individual and community health [1]. Every year in the USA, many people suffer from diseases that vaccines can prevent. Consequently, prioritizing the prevention of infectious diseases by increasing vaccination rates remains a public health priority. Despite this importance, there has been a notable decline in routine vaccinations, primarily driven by vaccine hesitancy [1].

Individual, social, economic, and environmental factors create challenges to vaccination access and uptake, often affecting racial and ethnic minorities and those living in communities that are economically underserved [2]. For instance, limited healthcare access, a lack of transportation, and medical mistrust have been found to contribute to vaccine disparities and hesitancy [3]. Thus, ensuring vaccine equity, access, and uptake for groups experiencing disparities in immunization requires addressing these inequities, social determinants of health (SDoH), and vaccine hesitancy [4]. Global pandemics further expose these inequities [4], compelling communities and the healthcare field to innovate sustainable solutions [1]. Individuals in the USA with lower incomes are less likely to receive the Influenza vaccine [5], while mistrust of the medical community among African Americans/Black contributed to the low uptake and demand for the H1N1 vaccine (swine flu) [6].

Research demonstrates the role of trust and community collaboration in addressing vaccination disparities and hesitancy [7]. Community-engaged strategies are pivotal to understanding and better meeting community vaccination needs to improve uptake and address hesitancy [2]. These strategies draw on trusted community-based organizations (CBOs) and community members’ experiences to meet vaccination needs effectively [1]. Thus, a university-public health partnership aligning with the T4 translational research stage [8] can improve health equity and mitigate vaccination disparities. Adding pharmacy personnel to these partnerships, leveraging their knowledge and expertise, can reduce disparities in the uptake of immunization services in community pharmacy settings [9].

Public health partners, including community pharmacies and community-based organizations (CBOs), have been pivotal in vaccine rollout. Community pharmacies offer convenient, high-quality, cost-effective healthcare services[10], contributing to administering over 305.5 million COVID-19 vaccine doses and addressing uptake and hesitancy [11,12]. Incorporating a trusted CBO partner improves access to systemically marginalized populations experiencing SDoH challenges and promotes community trust [13]. Leveraging academic health centers alongside CBOs has been found to address health disparities in rural communities successfully [14]. Despite these advancements, efforts have not incorporated a community pharmacy partner. Collectively, this evidence supports the development of a university-public health partnership to include community pharmacies to improve health care for people who are medically underserved [14].

The objective of this study was to describe and evaluate a university-public health use case that employed practical SDoH-based strategies to address vaccination disparities from June 2021 to October 2023. The evaluation utilized translational science benefits concepts to collate the use case processes and outcomes, positioning the findings as a transformative example of practical best practices for implementing SDoH-based strategies to improve health.

## Methods and materials

### Conceptual framework

**Figure 1.**
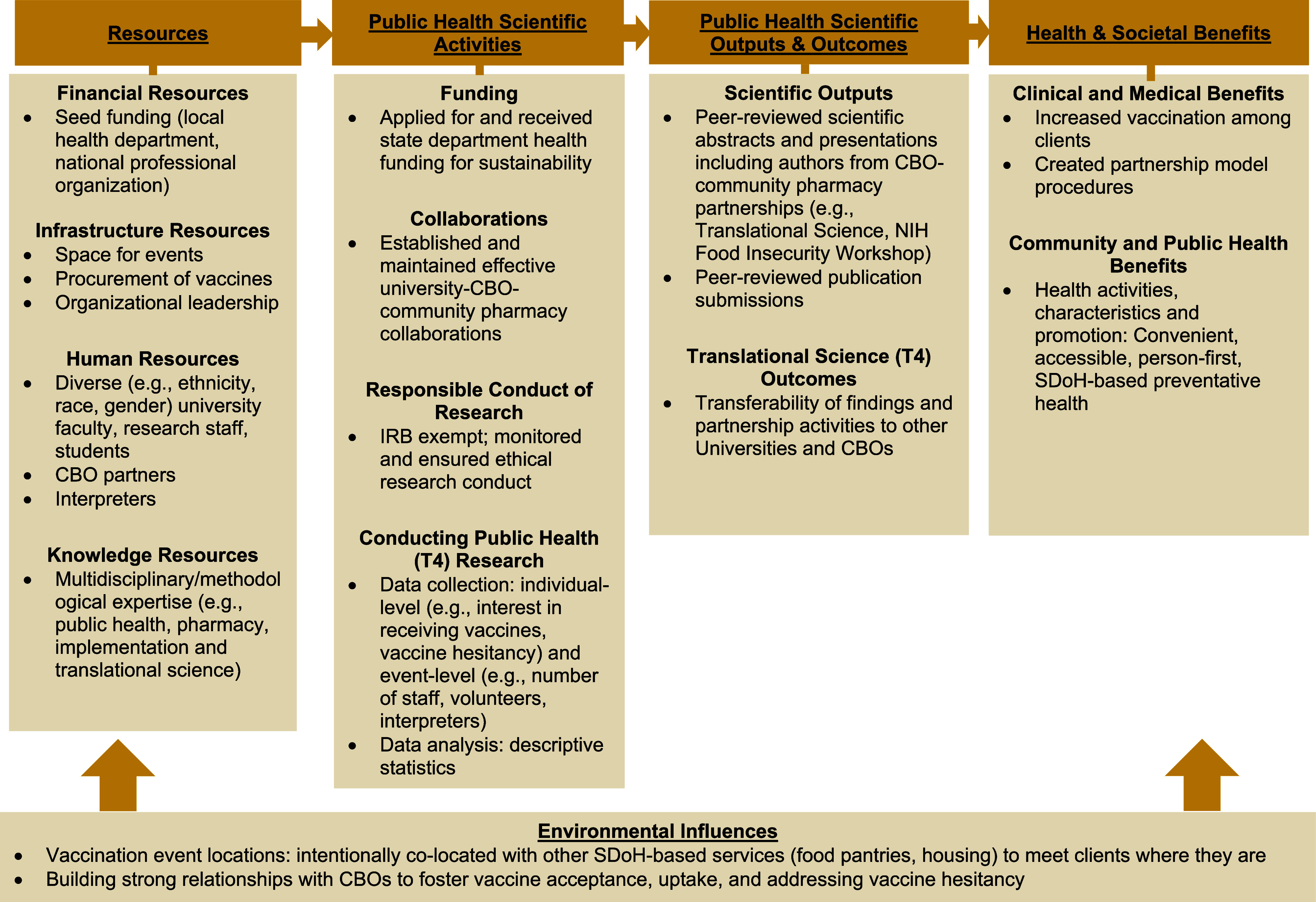
Translational science benefits logic model adapted for public health (T4) research. CBO = community based organization; SDoH = social determinants of health.

As recommended for clinical and translational research, we adapted the Translational Science Benefits Logic Model (TSBM) [15] to describe the university-public health partnership and subsequent evaluation. The TSBM illustrates how the partnership resources (e.g., finances, knowledge) guide and facilitate scientific activities (e.g., collaborations, conducting research) associated with the partnership. These activities link to scientific output, outcomes, and health and societal benefits, serving as criteria for assessing the impact of the partnership (Fig. 1).

### Use case partnership overview

In 2021, the Purdue University Center for Health Equity and Innovation (CHEqI) partnered with Gleaners Food Bank of Indiana and Walgreens to offer no-cost preventative vaccines (Influenza and COVID-19) at food bank distribution centers and mobile pantry sites. CHEqI, the university partner, is one of Purdue’s first health equity coordinating centers [16]. Initially, the partnership focused on Gleaners Food Bank, a major Midwest food bank with a shared interest in improving vaccine access. Walgreens, the community pharmacy partner, facilitated vaccine procurement. By late 2021, the partnership expanded to include St Vincent de Paul Food Pantry and Wheeler Mission, which provides services to individuals experiencing homelessness. These collaborations extended to other organizations, including community cultural centers, churches, and youth camps.

### Partnership and alignment to conceptual framework

The subsections describe a use case application of a university-CBO partnership guided by the adapted TSBM model (Fig. 1). The TSBM provides and guides scalable strategies for university-community partnerships across five primary domains: resources, public health scientific activities, outputs, and outcomes, as well as the ensuing health and social benefits. Through our use case example, we provide essential components and details for establishing university-CBO partnerships, offering insights into establishing and co-locating preventive and vaccination health services.

### Partnership resources

The partnership secured financial resources through seed funding from the Marion County Public Health Department and funds from the National Association of Chain Drug Stores. Grant resources supported pharmacy fellows, interpreters, incentives for student volunteers, advertising, and supplies. Infrastructure resources included event spaces, vaccine procurement, and organizational leadership, with the university partner overseeing logistics. Human resources involved diverse university faculty, research staff, students, and community partners. Knowledge resources leveraged multidisciplinary and methodological expertise from public health and pharmacy professionals to implement and translate science through vaccine events, evaluation, and dissemination.

### Public health scientific activities

Strategic funding and dynamic collaborations sustained the partnership. State Department Health funding supported ongoing activities, growth, and impact. The partnership harnessed collective strengths to address vaccination disparities and maximized expertise, resources, and communities to address complex vaccination-related challenges and barriers.

Regarding responsible conduct of research, this study received exempt approval from the Institutional Review Board and ensured ethical research practices. The university partner conducted public health (T4) research, collecting vaccine interest and outcome data. The partnership recognized the value of validated instruments in assessing vaccine interest and hesitancy, yet it was not the primary focus of these interactions. We deemed administering lengthy questionnaires that could disrupt the CBOs infeasible and impractical.

Trained event volunteers conducted guided conversations with CBO clientele to collect *vaccine interest data*. Volunteers have public health and pharmacy backgrounds, exposing them to special populations and minority groups. We trained volunteers by providing examples of common vaccine hesitancy reasons and effective talking points. Student volunteers with Spanish language skills connected effectively with the local Hispanic/Latino community. Volunteers had conversations to (1) determine the need for vaccination, (2) assess vaccination interest, and (3) address vaccine hesitancy through person-centered discussion. Volunteers asked clients about their vaccination interest and booster status, recorded responses on a questionnaire, and returned to a university point-person. Volunteers assessed vaccine hesitancy subjectively based on clients’ responses during the questionnaire administration. Volunteers addressed vaccine hesitancy by providing vaccination data, tailoring conversations to address specific concerns, and building trust with clientele through consistent presence and a diverse staff. We collected hesitancy data when sufficient volunteers were available (*n* = 44 events).

University staff collected additional information on individuals who received vacations to gather *vaccine outcome data*, including age, ethnicity, race, gender, and which vaccine(s) the client received via the Vaccine Administration Record. University staff collected event-level data, including the number of staff, volunteers, and interpreters present, along with the total number of clients who passed through the food bank/food pantry, and which vaccines were offered at the event.

We used IBM SPSS Statistics (Version 29) to compute descriptive statistics, characterizing program events and outcomes.

### Public health scientific outputs and outcomes

By leveraging existing and expanded scholarship stemming from this partnership, collaborators foresee its potential for replication across other Universities and CBOs, catalyzing future SDoH strategies and public health initiatives.

### Health and Societal Benefits

The partnership’s clinical and medical benefits include the number of vaccines administered and the clients’ demographic information. The partnership subsequently increased vaccine knowledge and decreased vaccine hesitancy by administering vaccines to CBO clientele. The intermediate partnership outcome is the sustainable, mutually beneficial collaboration between a university, community pharmacy, and CBOs as activities evolve and expand.

## Results

Table 1 includes findings of the public health partnership aligned with TSBM domains. One hundred seven vaccination events occurred from June 3, 2021, to October 27, 2023, with 91.6% occurring at food banks/pantries and homeless shelters. Most events occurred at indoor locations, and approximately 16.8% occurred at mobile food pantry events. Food pantries/banks served an average of 577 families per event over two to four hours.

**Table 1.** University-public health partnership vaccination event input characteristics, activities, outputs, and outcomes as defined by the logic model [15]

Vaccination Event Resources (*N* = 107)	Result
**Event Location**	***n* (%)**
Food Banks/Food Pantries	70 (65.4)
Homeless Shelters	28 (26.2)
Other (church, children’s camp, community center)	9 (8.4)
**Event Type**	***n* (%)**
Mobile Food Pantry Event	18 (16.9)
Indoor Event (*Food Bank, Homeless Shelter, and Other Event Sites)*	89 (83.2)
**Types of Preventative Vaccines Offered at Each Event**	***n* (%)**
One preventative vaccine	39 (36.6)
Two preventative vaccines	68 (63.6)
**Average Number of Staffing and Volunteers Per Event**	**Mean [SD], (Min–Max)**
Pharmacy Staff	1.5 [1.4], 1 (1–8)
University Staff	2.7 [1.4], 2 (0–7)
Volunteers	3.6 [2.9], 3 (0–15)
Hired Interpreters (Available at 17 of the 107 Events)	2 [0.94], 2 (0–4)
**Vaccine Event Public Health Activities, Outputs, and Outcomes**	**Result**
**Food Distribution at Food Bank/Food Pantry Events**	
Total # Households Served Across the Food Bank/Food Pantry Events, (*n*)	34,046
Average # of Families Served Across the Food Bank/Food Pantry Events, (Mean [SD], (Min–Max))	577.05 [253.93.9], 535 (11–1290)
**Baseline Characteristics of Individuals Receiving at Least One Vaccine**	
**^a^ Age *(N = 2432)***	**Mean [SD], Median (Min–Max)**
Average Age of Individuals Receiving at Least One Vaccine	43.98 [17.39], 44 (3–101)
**Sex *(N = 2438)***	***n* (%)**
Male	1236 (50.7)
Female	1202 (49.3)
** Race *(N = 2047)***	***n* (%)**
Black or African American	643 (31.4)
White	680 (33.2)
Other	596 (29.1)
American Indian or Alaskan Native	21 (1.0)
Asian	101 (4.9)
Native Hawaiian or Pacific Islander	6 (0.3)
** Ethnicity *(N = 2099)***	***n* (%)**
Latino	968 (46.1)
NOT Latino	1131 (53.9)
**Vaccine Interest and Administration**	
** Assessing Vaccine Interest** (Assessed at 57 of 107 events)	**Mean [SD], Median (Min–Max)**
Average # of People Assessed for Vaccine Interest Per Event	126.8 [88.9], 100 (7–310)
** Assessing Vaccine Hesitancy** (Assessed at 53 of 107 events)	**Mean [SD], Median (Min–Max)**
Average # of People Per Event that Were Hesitant to Receive a Vaccine	23.1 [20.7], 16 (1–92)
** Total Number of Vaccines Administered Across Events (*N* = 3021)**	
** Vaccines Administered Per Event**	**Mean [SD], Median (Min–Max)**
Average # of Vaccines Administered Per Event	28.2 [22.9], 22 (2–133)

a Age impacted by age-specific dosing recommendations for COVID-19 and influenza vaccines.

Events occurred within under-resourced areas in Indianapolis. Most vaccinated individuals self-identified as Black or African American (31.4%) or White (33.2%). Additionally, 46.1% of vaccinated individuals self-identified as Latino ethnicity and frequently necessitated interpreters who spoke Spanish for communication. While approximately 65% of events required interpreters, only 16% hired professional interpreters, and the remaining events had sufficient volunteers and staff serving as interpreters.

The majority of vaccination events provided two different preventative vaccines (63.6%). A total of 3,021 vaccines were given across all events, with a mean of 28 vaccines given per two- to four-hour event. One noteworthy food pantry event successfully administered 133 preventative vaccines. Volunteers assessed vaccine interest at 53% of events, with a mean of 127 people assessed per event. CBO clientele were also screened for vaccine hesitancy at 50% of events, with volunteers reporting that 1,225 individuals were vaccine-hesitant.

## Discussion

During the pandemic, affordability, poor healthcare experiences, language barriers, and transportation issues worsened healthcare access [3]. Aligned with the T3 and T4 translational pipeline, initiatives addressing these challenges across academic and community sectors offered the potential for greater impacts on individual and community health [17]. This public health partnership focused on addressing vaccination access disparities among racial/ethnic minorities and marginalized populations by co-locating vaccination services with SDoH services to ensure convenient access. In partnering with CBOs providing services to food and/or housing-insecure populations, the partnership utilized person-centered conversations to address vaccine hesitancy. Effective communication, providing clear and accurate information on vaccine safety and benefits, has been found to mitigate vaccine hesitancy and improve vaccine confidence [18].

The university and Walgreens staff’s consistent presence at CBO sites contributed to addressing vaccine hesitancy and increasing uptake. Over time, as clients receive new information, their views of vaccination may change. This approach aligns with patient reminder interventions and evidence-based strategies that have shown success in increasing immunization uptake and reducing hesitancy [19].

While barriers to vaccination persist, there is a decline in pandemic response funding, demonstrating a need for an efficient and cost-effective model for providing vaccinations. This model effectively co-locates vaccinations with other services, serving as a vaccination and population health strategy that complements prevention efforts such as point-of-care testing and health screenings.

This public health partnership model has expanded to provide additional health services, including naloxone distribution, blood pressure screenings, and tobacco cessation education. These efforts have shown early promise in initial data. University-CBO partnerships bringing vaccinations and other preventive health services to communities that are under-resourced effectively reduce access barriers and address health disparities. Providing interpreters, as recommended by the CDC [20]. enhances health literacy for diverse populations.

This partnership model, successful in urban settings, has begun pilot vaccination events in rural areas. Initial findings indicate the need to tailor the model to be relevant to rural communities, including understanding cultural norms and building trust, particularly within faith-based communities like the Amish.

### Limitations

This evaluation focused on enhancing vaccine uptake and addressing vaccine hesitancy among individuals experiencing food insecurity and homelessness while minimizing disruptions to food distribution and shelter operations. However, the study has limitations. The University partner tailored data collection strategies to prevent disruptions, limiting the use of more sophisticated evaluation methods and validated instruments for assessing vaccine hesitancy. Due to privacy concerns, events did not collect patient-identifiable data, impeding the tracking of repeat vaccinations at events. These limitations highlight the need for future research to collect more comprehensive data while considering operational demands when engaging with CBOs.

## Conclusion

The public health partnership demonstrated success in efficiently administering preventative vaccines, making it a model applicable to numerous health disparities prevalent in communities that are under-resourced. Future research can evaluate the longer-term impact on health and healthcare optimization while also investigating the experiences of key collaborators (e.g., university personnel, students, community partners, and community pharmacies) that are integral to fostering and sustaining the partnership.
